# 

**Published:** 1900-03

**Authors:** 


### CONTENTS.

ORIGINAL COMMUNICATIONS.

Filling-Materials. By C. N. Johnson, L.D S., D.D.S. . . . . . . . . . . . 145
Is it Possible to Jump the Bite? By Eugene S. Talbot, M.D., D.D.S. . . . . 158
The Use of Compressed Air in Operative Dentistry. By Dr. S. Freeman . . . 162
Removal of Superior Maxilla and Preservation of Facial Expression. By Dr. C. B. Parker . . . . . . . . . . . . . . . . . . . . . . . . . . 169

ABSTRACTS AND TRANSLATIONS.

The Antiseptic and Disinfectant Properties of Soap . . . . . . . . . . . 171

REPORTS OF SOCIETY MEETINGS.

National Dental Association . . . . . . . . . . . . . . . . . . . 174
The New York Institute of Stomatology . . . . . . . . . . . . . . . 181
American Academy of Dental Science . . . . . . . . . . . . . . . . 190
Academy of Stomatology . . . . . . . . . . . . . . . . . . . . . 202

EDITORIAL.

Dental Literature of the Past and Present. . . . . . . . . . . . . . 208

DOMESTIC CORRESPONDENCE.

Tempering in Glass Tubes . . . . . . . . . . . . . . . . . . . . 213
Reply to Dr. B. S. Scott, on the Use of "Volasem" . . . . . . . . . . 213

OBITUARY.

Meeting of American Graduates at Sydney, New South Wales . . . . . . . 215

CURRENT NEWS.

International Dental Congress . . . . . . . . . . . . . . . . . . 216
Board of Dental Examiners, Commonwealth of Pennsylvania . . . . . . . . 218
Massachusetts Board of Registration in Dentistry . . . . . . . . . . . 219
Missouri State Board of Dental Examiners . . . . . . . . . . . . . . 219

